# Annual screening of moderate-risk women: a review of 10 years experience within the NHS Breast Screening Programme

**DOI:** 10.1186/bcr3290

**Published:** 2012-11-09

**Authors:** ND Forester, CE Holmes, N Sibal, L McLean

**Affiliations:** 1 Department of Breast Radiology, Newcastle upon Tyne, UK

## Introduction

We have offered annual breast screening to women under 50 years with moderate risk of breast cancer (as defined by NICE guidelines) within the NHS Breast Screening Programme in Newcastle for over 10 years. We evaluated the process and outcome of this programme in this subpopulation of screened women.

## Methods

A retrospective review of women screened between April 2002 and March 2012. The population screened, screening episodes, recall rate and outcomes were assessed.

## Results

A total of 1,027 moderate risk women were screened over 10 years, resulting in 5,406 screening episodes generated. There were 135 recalls from screening in 126 women, giving a recall rate of 2.5%. Of the 135 recall episodes, 100 were benign on further imaging and clinical examination. Thirty-five underwent biopsy, of which 13 were malignant. This gives a cancer detection rate of 2.4 per 1,000 women screened.

## Conclusion

Screening of moderate-risk women is possible within the NHS Breast Screening Programme. The recall rate of 2.5% falls within the acceptable standards for both prevalent and incident screens set in the over-50 years population. As this is annual screening, the overall cancer detection rate is similar to that found in the over-50 years group (approximately 6 per 1,000 over 3 years). Screening is therefore as effective in this increased risk population as the current NHS Breast Screening programme is for over-50 year olds.
